# Toward TiO_2_ Nanofluids—Part 2: Applications and Challenges

**DOI:** 10.1186/s11671-017-2185-7

**Published:** 2017-07-06

**Authors:** Liu Yang, Yuhan Hu

**Affiliations:** 1 Key Laboratory of Energy Thermal Conversion and Control of Ministry of Education, School of Energy and Environment, Southeast University, Nanjing, China; 2 Jiangsu Provincial Key Laboratory of Solar Energy Science and Technology, School of Energy and Environment, Southeast University, Nanjing, China

**Keywords:** Nanofluids, Thermal conductivity, Application, Solar absorption

## Abstract

The research about nanofluids has been explosively increasing due to their fascinating properties in heat or mass transportation, fluidity, and dispersion stability for energy system applications (e.g., solar collectors, refrigeration, heat pipes, and energy storage). This second part of the review summarizes recent research on application of TiO_2_ nanofluids and identifies the challenges and opportunities for the further exploration of TiO_2_ nanofluids. It is expected that the two exhaustive reviews could be a helpful reference guide for researchers to update the knowledge on research status of TiO_2_ nanofluids, and the critical comments, challenges, and recommendations could be useful for future study directions.

## **Review**

### Background

In the first part, the studies on the preparation, stability, and properties have been reviewed. It can be seen that many researches have been carried out on the directions of preparation and properties of nanofluids [1–7]. Meanwhile, there are also many attempts that have been made for application of nanofluid, especially in energy systems [8–11]. Due to the enhancement in heat and mass transfer process, TiO_2_ nanofluids have been tentatively applied to the fields of solar collectors [12], refrigeration [13–16], energy storage [17, 18], heat pipes [19–21], and other energy applications [22–34], such as car radiator [31], PV/T hybrid system [32, 33], and combined heat and power (CHP) systems [34]. In our previous studies, the heat transfer characteristics of TiO_2_ nanofluids in heat conduction, forced convection boiling heat transfer, and natural convection heat transfer have been summarized [35]. However, it is far from a comprehensive summary for application of TiO_2_ nanofluids; there are also many practical applications for TiO_2_ nanofluids. Here, in part 2, we will provide a detailed review on the thermal conductivity and energy-related applications of TiO_2_ nanofluids. We hope that the two reviews combined with our previous report [35] can provide a comprehensive understanding on research progress of TiO_2_ nanofluids. With the development of nanofluid technology, it is expected that nanofluids will be practically applied as a new and efficient work fluid for those energy systems.

## Application in Enhancing the Thermal Conductivity

Since the outstanding performance of nanofluids is generally attributed to the physical properties of fluids with the addition of nanoparticles, the experimental or theoretical investigations on the thermal conductivity of nanofluids should be an important topic in the field of nanofluids. Although most review articles introduced the thermal conductivity in the physical property part, enhancing the thermal conductivity is also an important application aspect of nanofluids. Another reason of putting thermal conductivity in the application part is to balance the content of the two reviews.

Many experimental and theoretical research results have shown that the addition of nanoparticles can distinctly improve the thermal conductivity of fluid. The influence factors on the thermal conductivity of nanofluids can be induced as the following groups: (1) internal factors, including particles’ type, content [36, 37], size [38], shape [39], and structure [40] and type of base fluid [41] and probable surfactant or pH regulator [42, 43] if have; (2) external factors, including temperature [40], supersonic vibration time [44], storage time [45], or running time [46]; and (3) microcosmic factors, such as surface charge state of nanoparticles [47], cluster of particles [48], the interfacial nanolayer [49], Brownian motion [50], the aggregation [51], interfacial thermal resistance, and mass difference scattering [52]. Our previous study has provided a table to show the thermal conductivity of TiO_2_ nanofluids [35]. However, it is not intuitive and inconvenient to understand the different effect factors on the influence degree. Therefore, in this part 2, the influences on the thermal conductivity of TiO_2_ nanofluids are shown in figures to provide a more perceptual understanding.

### Particle Loading Effect

**Fig. 1:**
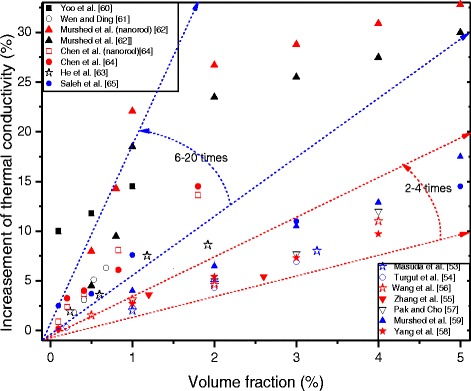
Volume fraction dependence of thermal conductivity of TiO_2_–water nanofluids in available literatures

A summary of the increment of the thermal conductivity of TiO_2_–water nanofluids with the volume fraction of nanoparticles in available literatures is shown in Fig. 1. It can be seen from all experimental results that TiO_2_ nanoparticles can enhance the thermal conductivity of base fluids. However, the increments of different researches are profoundly different. For instance, one enhancement on the thermal conductivity of nanofluids is about 2–4 times of volume loading of TiO_2_ nanoparticles, including Masuda et al. [53], Turgut et al. [54], Zhang et al. [55], Wang et al. [56], Pak and Cho [57], Yang et al. [58], and Mushed et al.’s [59] results. The other enhancement can reach 6–20 times of volume loading of TiO_2_ nanoparticles, including Yoo et al. [60], Wen and Ding [61], Mushed et al. [62], He et al. [63], Chen et al. [64], and Saleh et al.’s [65] results.

The differences of results are probably as a result of, besides the volume fractions, the thermal conductivity of TiO_2_ nanofluids is also determined by the particles’ parameters and the environmental circumstances, such as particle size and shape, surfactant, pH value, and temperature, which were quite different in different works. Moreover, some researchers observed that nanoparticles have little effect on the thermal conductivity of TiO_2_ nanofluids. Utomo et al. [66] investigated the thermal conductivity of water-based alumina and titania nanofluids. They observed that the thermal conductivity of TiO_2_ nanofluids they prepared was slightly lower than the conventional model prediction due to the high content of dispersants. And the results clearly showed that TiO_2_ nanofluids do not show anomalously thermal conductivity enhancement or convection heat transfer coefficient in a pipe flow as showed in other reports.

### Particle Shape Effect

The influences of shape and size of nanoparticles are not as widely investigated as that of particle loading. The existing studies have not shown great effects by the particle shape or size on the thermal conductivity of TiO_2_ nanofluids, which is most probably due to the relatively small quantities on this effect. Murshed et al. [62] dispersed two kinds of TiO_2_ nanoparticle water using CTAB as dispersant. One type is in rod-shape with a diameter by length of 10 nm × 40 nm. And the other type is in spherical shapes of 15 nm in diameter. They observed that the thermal conductivity of both kinds TiO_2_ nanofluids increased with the increase in particle loading, while the rod-like particles had more contributions than spherical ones. The maximum enhancements in the thermal conductivity for the former and the latter were about 33 and 30%, respectively. Chen et al. [64] studied the effective thermal conductivity of four types of nanofluids orthogonally made of TiO_2_ nanoparticles (25 nm) and TiO_2_ nanotubes (10 nm × 100 nm) with water and EG as base fluid, respectively. They found that the distinctions between the enhancement of TiO_2_ nanoparticles and TiO_2_ nanotubes on the thermal conductivity were not large, while the enhancement is much larger than the calculation value of Hamilton–Crosser equation.

### Temperature Effect

**Fig. 2:**
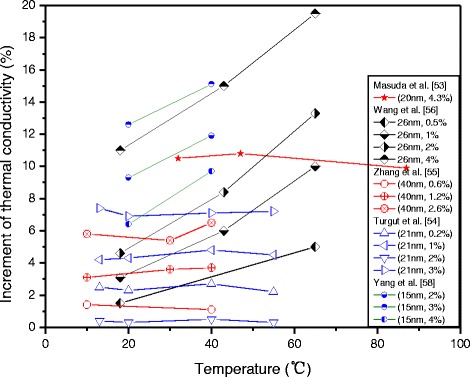
Influence of temperature on the enhancement of thermal conductivity of TiO_2_ nanofluids in different researches

Temperature is another important influence factor on the thermal conductivity of TiO_2_ nanofluids. Figure 2 shows the influence of temperature on the enhancement of thermal conductivity of TiO_2_ nanofluids in different researches. Wang et al. [67] investigated the effect of particle loading and temperature on the thermal conductivity of water-based TiO_2_ nanofluids. The results showed that the working temperature plays more important positive roles and makes more contribution to the thermal conductivity at a higher temperature. They also concluded that the results agreed with the theoretical values determined by considering the temperature-dependent Brownian motion and the micro-convection. Reddy et al. [68] investigated the thermal conductivity of TiO_2_ nanofluids for different particle loading in the range of 0.2–1.0% at different temperatures. And they observed that thermal conductivity of TiO_2_ nanofluids increases with an increase in both particle loading and temperature. Yang et al. [58] added TiO_2_ nanoparticles to ammonia–water to prepare binary fluid-based nanofluids. They also found that the increase in temperature could result in an increase in the thermal conductivity ratio of binary TiO_2_ nanofluids to base fluid.

The above results showed that TiO_2_ nanoparticles can make more contribution to the thermal conductivity of TiO_2_ nanofluids at higher temperature. However, some singular results about the effect of temperature can be also included. Turgut et al. [54] investigated the effective thermal conductivity of deionized water-based TiO_2_ nanofluids at temperatures of 13, 23, 40, and 55 °C. They observed that the thermal conductivity increases with an increase in particle loading but the change of temperature has little effect on the effective thermal conductivity of TiO_2_ nanofluids. In addition, some results showed that temperature plays roles on the effective thermal conductivity. Duangthongsuk and Wongwises [69] suspended TiO_2_ nanoparticles in water with a volume loading range of 0.2 to 2%, and they collected the data at a temperature range of 15 to 35 °C. They observed that the measured thermal conductivity of TiO_2_–water nanofluids increased with the increase in both particle loading and temperature, but the thermal conductivity ratio decreased when the temperature increased; they attributed the reason to the faster growth rate of thermal conductivity of base fluid.

**Fig. 3:**
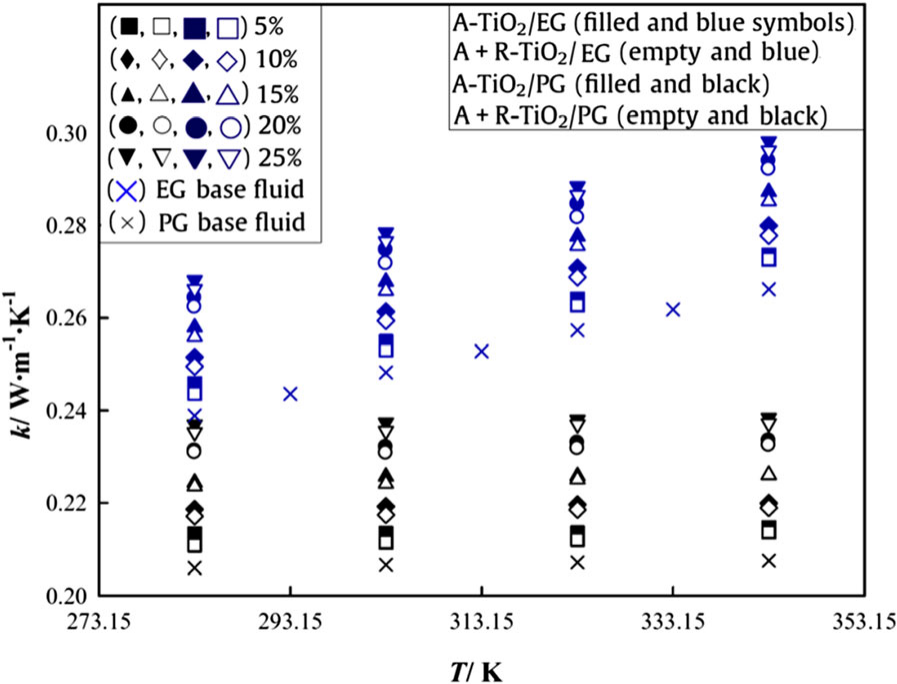
Temperature dependence of the thermal conductivity of four kinds of TiO_2_ nanofluids [41]. Reproduced with permission from Elsevier

The reason of the uncertain role of temperature on the thermal conductivity ratio of TiO_2_ nanofluids may be due to the complex mechanism of thermal conductivity of nanofluids. When temperature changes, the other parameters, such as the structure, surface activity, stability and of particles, the characteristic of dispersant, etc. may be changed, and those parameters are generally much different in different works. Therefore, the influences of temperature on the thermal conductivity ratio of TiO_2_ nanofluids are related to the specific nanoparticles and base fluid types. This observation can be further improved by Cabaleiro et al.’s research [41], in which the temperature-dependent thermal conductivity behavior was studied for anatase and rutile TiO_2_ nanofluids with ethylene and propylene glycol as base fluid, respectively. The temperature dependence of the thermal conductivity of these four kinds of TiO_2_ nanofluids is shown in Fig. 3. It can be observed that all the four kinds of nanofluids exhibited higher thermal conductivities than the corresponding base fluids. Temperature played different roles for TiO_2_ nanofluids containing different nanocrystalline structure nanoparticles and with different base fluids. The thermal conductivity increased as temperature increases for EG-based nanofluids, with a maximum increment of 11.4% by the temperature in the study range, while it seemed almost independent of temperature for PG-based nanofluids.

### Base Fluid Effect

Ingredients of base fluids can also affect the thermal conductivity of TiO_2_ nanofluids. Chen et al. [64] measured the effective thermal conductivity of spherical and tubular TiO_2_ nanofluids with water and ethylene glycol as base fluids, respectively. They observed that both of the enhancements of TiO_2_ nanoparticles and TiO_2_ nanotubes with EG as base fluids were higher than that with water as base fluid. Reddy et al. [68] found that the thermal conductivity enhancement for water-based, EG/W (40%:60%)-based, and EG/W (50%:50%)-based TiO_2_ nanofluids increased from 0.649 to 5.01%, 1.94 to 4.38%, and 10.64 to 14.2%, respectively, when the volume concentration of TiO_2_ nanoparticles increased from 0.2 to 1.0% at room temperature (30 °C). However, some opposite results can also be observed, Cabaleiro et al. [41] found that the thermal conductivity enhancements for TiO_2_ nanofluids with EG, PG, or paraffin oil as base fluids were distinctly lower than those with water as base fluids. Also, in Sonawane et al.’s report [70], the effect of base fluids was thought to be complex and inaccessible because the thermal conductivity of TiO_2_ nanofluids with 1 vol.% particle loading followed the following sequence: paraffin oil-based nanofluid > water-based nanofluid > EG-based nanofluid, while that of pure base fluids followed the sequence water > EG > paraffin oil. They analyzed this erratic observation from the perspective of the viscosity effect and thought that lower base fluid viscosity could make more contributions to the enhancement of the thermal conductivity of nanofluids.

### Surfactant Effect

**Fig. 4:**
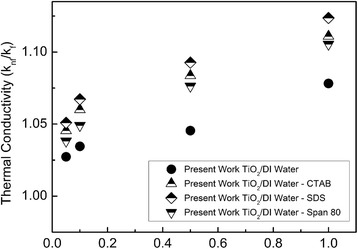
Effect of different surfactants on the thermal conductivity of TiO_2_–water nanofluids [65]. Reproduced with permission from Elsevier

The addition of surfactant is another important factor on the thermal conductivity of TiO_2_ nanofluids. Some results showed the surfactants have a positive effect on thermal conductivity. Saleh et al.’s [65] studied the effect of different types of surfactants on the thermal conductivity of TiO_2_–water nanofluids, and the results are shown in Fig. 4. It can be seen that all of the three kinds of surfactants could greatly improve the thermal conductivity of nanofluids and the nanofluids with SDS as stabilizer exhibited the greatest enhancement, followed by those with CTAB and Span-80 as stabilizer. And they thought that the dispersion stability and surface properties of the particles was involved in the enhancements in thermal conduction of nanofluids.

There are also some different results on the surfactant effect. Yang et al. [58] found that when the content of ammonia in base fluids increases, the thermal conductivity ratio of TiO_2_ nanofluids will also increase because the stability of TiO_2_ ammonia–water nanofluids will be improved in higher pH value. And the surfactants PEG1000 and PAA in low concentration have relatively smaller influence than other impact factors on thermal conductivity like, particles or ammonia content, temperature. However, PEG1000 can improve the stability TiO_2_ ammonia–water nanofluids, which induce the improvement of thermal conductivity of nanofluids. Murshed et al. [62] found that oleic acid and CTAB can improve the dispersion stability of TiO_2_ nanofluids without impacting on thermal physical properties of nanofluids and single-phase heat transfer coefficient because the surfactant content employed in their experiments was very low viz. 0.01–0.02 vol.%. There are also some results that showed the surfactants have a depressing effect. Utomo et al. [66] investigated the thermal conductivity of water-based Al_2_O_3_ and TiO_2_ nanofluids. They found that high loading of stabilizers could result in a decrease in the effective thermal conductivity of those two kinds of nanofluids.

### Sonication Effect

**Fig. 5:**
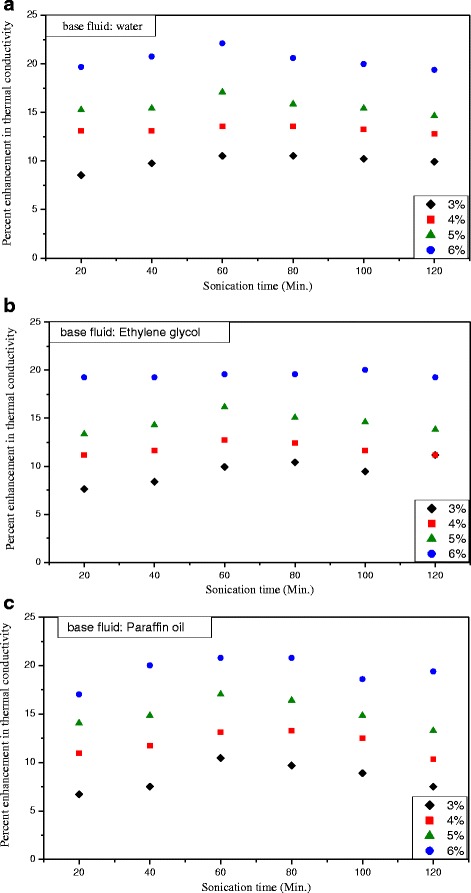
The percent enhancement in thermal conductivity as a function of sonication time. **a** Base fluid: water. **b** Base fluid: ethylene glycol. **c** Base fluid: paraffin oil. Redrawn based on experimental data in reference [70]

The sonication also showed some effects on the thermal conductivity of TiO_2_ nanofluids. Ismay et al. [71] found that the thermal conductivity of TiO_2_–water nanofluids achieved the maximum when the pH value is close to 7 and was further improved by 2 h’ sonication. And they thought that aggregation can explain the observed enhancements due to the percolation effect. Sonawane et al. [70] performed a particular research about the effect on thermal conductivity by the ultrasonic time, and the results are shown in Fig. 5a–c. It can be found for all the three kinds of nanofluids in various concentrations, the increasing proportions of thermal conductivity increased firstly and then decreased as the ultrasonic time increases, and the maximum increment occurred at the sonication time of 60 min. They attributed the reason as follows: optimum sonication time of 60 min can intensify the Brownian motion of nanoparticles and the intermolecular interaction between particles and bulk liquid, which resulted in an enhancement of thermal conductivity. However, longtime sonication of exceeding 60 min could induce the clustering and aggregating of nanoparticles, which was thought accountable for the decline of heat transport and thermal conductivity in nanoparticles.

### Theoretical Studies

The theoretical study of nanofluids is one of the research hotspots in the field of nanofluids. There have been numerous thermal conductivity models proposed in recent years. It is generally regarded that most conventional models can be used for TiO_2_ nanofluid unless there are special restrictions. However, due to the great difference in the experimental data of thermal conductivity of TiO_2_ nanofluids, it is almost impossible for a single model to fit all different results. Due to the conventional models are hard to be applied to an individual case, some targeted thermal conductivity models for TiO_2_ nanofluids are also proposed in recent years. Table 1 provides a summary of available thermal conductivity model equations specialized in TiO_2_ nanofluids. It can be seen that the factors such as interfacial layer [59, 72], Brownian movement [73, 74], particle size and aspect ratio [72, 75], and aggregation [76] have been considered in some models. And there are also some models that are proposed just by experimental fitting or analysis of variance [68, 74, 77–82]. It can be possible to conclude that those models are only suitable for their individual cases. Although the theoretical studies on the thermal conductivity of nanofluids have been much developed, the most fundamental flaw lies in the great difference in different experimental results. It is rather difficult to comprehensively and accurately grasp the heat conduction process in nanofluid since the nanostructure and micromotion of particles are hard to quantitatively describe. Therefore, due to the poor accuracy of the models for an individual application case, the best way to obtain the thermal conductivity of nanofluids for designing the application system is to carry out a preliminary experiment.

**Table 1:** Theoretical expressions of existing thermal conductivity models for TiO_2_ nanofluids

Authors	Year	Model expressions	Note
Murshed et al. [59]:	2008	${k}_{\mathrm{eff}}=\frac{\left({k}_p-{k}_{lr}\right){\varphi}_p{k}_{lr}\left(2{\beta}_1^3-{\beta}^3+1\right)+\left({k}_p+2{k}_{lr}\right){\beta}_1^3\left[{\varphi}_p{\beta}^3\left({k}_{lr}-{k}_f\right)+{k}_f\right]}{\beta_1^3\left({k}_p+2{k}_{lr}\right)-\left({k}_p-{k}_{lr}\right){\varphi}_p\left({\beta}_1^3+{\beta}^3-1\right)}$ *β*_1_ = 1 + *t*/*R* *β*_2_ = 1 + *t*/(2*R*) *k*_*lr*_ and *t* are thermal conductivity and thickness of the interfacial layer.	This model considers the interfacial layer and has been validated for TiO_2_, Al_2_O_3_, and Al nanofluids.
Duangthongsuk and Wongwises [77]	2009	*k*_*nf*_ = *k*_*f*_(*a* + *bφ*) At 15 °C: *a* = 1.0225, *b* = 0.0272 At 25 °C: *a* = 1.0204, *b* = 0.0249 At 35 °C: *a* = 1.0139, *b* = 0.0250	It is a fitted linear equation for TiO_2_ nanofluids within 2 vol.%.
Corcione [73]	2011	$\frac{k_{\mathrm{eff}}}{k_f}=1+4.4 R{e}_p^{0.4}{ \Pr}_f^{0.66}{\left(\frac{T}{T_{f r}}\right)}^{10}{\left(\frac{k_p}{k_r}\right)}^{0.03}{\varphi}^{0.66}$ $R{e}_d=\frac{u_B{d}_p}{\upsilon}=\frac{2{\rho}_f{k}_B T}{\pi {\mu}_f^2{d}_p}$ where *T*_*fr*_ are the freezing point of the base fluid (about 273.16 K for water), *Re*_*p*_ is the nanoparticle Reynolds number.	This model considers Brownian motion and has been validated for TiO_2_, Al_2_O_3_, and CuO nanofluids. Applied range: 0.2% < φ < 9%, 10 nm < *d* < 150 nm, 294 K < *T* < 324 K.
Okeke et al. [76]	2011	(1 − *φ*_*nc*_)(*k*_*f*_ − *k*_*nc*_)/(*k*_*f*_ + 2*k*_*nc*_) + *φ*_*nc*_(*k*_*p*_ − *k*_*nc*_)/(*k*_*f*_ + 2*k*_*nc*_) = 0 where *k*_*nc*_ is the thermal conductivity of the imaginary medium with backbones. *φ*_*nc*_ is taken as the volume fraction of the particles which belong to dead ends.	This model considers the aggregate sizes, particle loading, and interfacial resistance based on fractal and chemical dimensions. And it has been validated for Al_2_O_3_, CuO, and TiO_2_ nanofluids.
Azmi et al. [78]	2012	$\frac{k_{\mathrm{eff}}}{k_f}=0.8938{\left(1+\frac{\varphi}{100}\right)}^{1.37}{\left(1+\frac{T}{70}\right)}^{0.2777}{\left(1+\frac{d}{150}\right)}^{-0.0336}{\left(\frac{\alpha_f}{\alpha_p}\right)}^{0.01737}$	This model has been validated for water based Al_2_O_3_, ZnO, and TiO_2_ nanofluids. Applied range: *φ* < 4%, 20 nm < *d* < 150 nm, 293 K < *T* < 343 K.
Reddy and Rao [88]	2013	*k*_*nf*_ = *k*_*f*_(*a* + *bφ*) Regression constants *a* and *b* at various temperatures for water, 40%:60% and 50%:50% EG/W.	It is a fitted expression for TiO_2_ nanofluids. Applied range: 30 °C < *T* < 70 °C, 0.2% < *φ* < 1%.
Zerradi et al. [79]	2014	*k*_*nf*_ = *k*_*s*_ + *k*_*b*_ ${k}_s=\frac{\frac{k_p}{k_f}+\psi +\psi \varphi \left(1-\frac{k_p}{k_f}\right)}{\frac{k_p}{k_f}+\psi +\varphi \left(1-\frac{k_p}{k_f}\right)}$ ${k}_B= G\frac{k_f{\varepsilon}_f{\displaystyle \sum_{i=1}^N\frac{A_i{ \Pr}^p}{d_i}\left[\alpha \varphi +\left(\beta +\chi \varphi \right){\left(\frac{1}{\nu}\sqrt{\frac{18{k}_b T}{\pi \rho {d}_i}}\right)}^q+\delta \right]}}{ \Pr {A}_T}$ where *ψ* is a shape factor defined by $\psi =2{\varphi}^{0.2}\frac{l_p}{d_p}$ for cylindrical particles *ψ* = 2*φ* ^0.2^ for spherical particles *α*, *β*, and *χ* are thermophysic coefficients.	This model is based on the Monte Carlo simulation combined with a new Nusselt number correlation. It has been validated for Al_2_O_3_–H_2_O, CuO–H_2_O, TiO_2_–H_2_O, and CNT–H_2_O nanofluids.
Abdolbaqi et al. [80]	2016	$\frac{k_{\mathrm{eff}}}{k_f}=1.308{\left(\frac{\varphi}{100}\right)}^{0.042}{\left(\frac{T}{80}\right)}^{0.011}$	It is nonlinear model for BioGlycol/water-based TiO_2_ nanofluids based on the aggregation theory using analysis of variance. Applied temperature range: 30 °C < *T* < 80 °C.
Shukla et al. [74]	2016	$\frac{k_{nf}}{k_f}=\left(1-\varphi \right)+\pi {\left(\frac{6}{\pi}\right)}^{1/3}{\varphi}^{4/3}{\left[\frac{1+0.5{\left(\frac{6\varphi}{\pi}\right)}^{1/3}}{2}\left(\frac{k_{bf}}{k_p}\right)+\frac{\psi}{Nu}\right]}^{-1}$ where *ψ* is the sphericity of nanoparticle.	This model considers Brownian motion. And it has been validated for water and EG-based TiO_2_ and Al_2_O_3_ nanofluids.
Wei et al. [81]	2017	100(*k*_*nf*_ − *k*_*nf*_)/*k*_*f*_ = 0.443 + 2.636*φ*	It is a linear fit of the measured values for diathermic oil-based TiO_2_ nanofluids. Applied range: *φ* < 1%.
Pryazhnikov et al. [82]	2017	$\frac{k_{nf}}{k_f}=1+4.82\varphi -23.1{\varphi}^2$	It is a fitted expression based on the measured values for 150 nm particles of TiO_2_.
Yang et al. [75]	2017	${k}_{\mathrm{eff}}=\frac{1}{\pi}{\displaystyle \underset{0}{\overset{\pi}{\int }}{\left({k}_z^2{ \sin}^2\omega +{k}_x^2{ \cos}^2\omega \right)}^{1/2} d}\omega$ ${k}_z=\frac{R{k}_p{k}_f}{\left( H+ R\right)\left({k}_p-\varphi {k}_p+\varphi {k}_f\right)}+\frac{ H\varphi {k}_p+ H\left(1-\varphi \right){k}_f}{H+ R}$ ${k}_x={k}_f\frac{k_p+{k}_f+\varphi \left({k}_p-{k}_f\right)}{k_p+{k}_f-\varphi \left({k}_p-{k}_f\right)}$ where* k*_*x*_ and *k*_*z*_ are the effective thermal conductivity in radial and axial directions, respectively.	This model considered the particle aspect ratio and has been validated for cylindrical TiO_2_ and Bi2Te3 nanofluids.
Yang et al. [72]	2017	${k}_{\mathrm{eff}}=\frac{\left( H+2 t\right){k}_{\mathrm{eff}\_ x}+\left( R+ t\right){k}_{\mathrm{eff}\_ z}}{H+ R+3 t}$ ${k}_{\mathrm{eff}\_ x}=\frac{A{\varphi}_p{k}_p+\left(\alpha B+\beta C\right){\varphi}_p{k}_{lr}+\left(1+\alpha +\beta \right){\varphi}_p{k}_f-{k}_f}{A{\varphi}_p+\left(\alpha B+\beta C\right){\varphi}_p+\left(1+\alpha +\beta \right){\varphi}_p-1}$ ${k}_{\mathrm{eff}\_ z}=\frac{H+2 t}{H}{\varphi}_p\left(\frac{\left( H+2 t\right){k}_{lr}{k}_p}{2 t{k}_p+ H{k}_{lr}}+\alpha {k}_{lr}\right)+{k}_f-\frac{\left( H+2 t\right)\left(1+\alpha \right){k}_f{\varphi}_p}{H}$ where $\alpha =\frac{{\left( R+ t\right)}^2-{R}^2}{R^2},$ $\beta =\frac{2 t{\left( R+ t\right)}^2}{H{ R}^2},$ $t=\sqrt{2\pi}\sigma$ $A=-\frac{2{k}_{lr}}{k_p+{k}_{lr}},$ $B=\frac{R}{R+ t}\cdot \frac{k_p-{k}_{lr}}{k_{lr}+{k}_p}-1,$ $C=-\frac{2{k}_f}{k_{lr}+{k}_f}$ ${k}_{lr}=\frac{k_p{\left(1+ t/ R-{k}_f/{k}_p\right)}^{{}^2}}{\left[\right(1+ t/ R-{k}_f/{k}_p-\left( t{k}_f\right)/\left( R{k}_p\right)\Big] \ln \left[\left(1+ t/ R\right){k}_p/{k}_f\right]+\left(1+ t/ R-{k}_f/{k}_p\right) t/ R}$	This model considered the interfacial layer and particle shape and has been validated for rod-like TiO_2_ and Bi2Te3 nanofluids.

The above analysis reveals that at present, there still exist controversies and inconsistencies on the influence factors on thermal conductivity of TiO_2_ nanofluids. Although particle loading has exhibited a positive correlation with thermal conductivity of nanofluids, the effects of other factors including particle shape, size, base fluid type, temperature, surfactant, and sonication are unified. Even for particle loading effect, the intensities of growth in thermal conductivity differ widely for different samples. The inconsistencies of thermal conductivity of nanofluids in various researches are mainly because the thermal conductivity is simultaneously affected by many factors especially some microscopic parameters such as particle clustering and micromotion which are rather difficult for a quantitative analysis or measurement.

Another controversy is the mechanism of enhancement in heat conduction of nanofluids. The particle clustering and gathering are thought to be responsible for the enhancement in the heat conduction of nanofluids [48, 50, 51]. However, the stable nanofluids with fewer aggregations by suitable surfactant or sonication treatment also have showed higher thermal conductivity [62, 65, 66, 70, 71]. The main mechanism of enhancement in heat conduction of nanofluids is the particle clustering or the micromotion, or some other factors need to be further analyzed.

## Solar Absorption

As a clean source of renewable energy, solar energy has the minimal environmental impact. However, the development of solar thermal collector is restricted by the poor absorption properties of the conventional working fluid. Therefore, in recent years, the nanofluid technology has been gradually used in the solar collectors to produce superior thermal and optical properties. It is expected that this new generation of heat transfer and solar absorption fluid can improve the efficiency of use of solar energy.

**Fig. 6:**
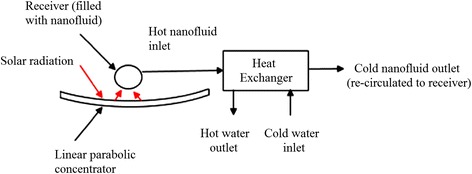
Schematic of nanofluid-based concentrating solar water heating system. Redrawn based on reference [83]

As shown in Fig. 6, a typical schematic diagram of nanofluid-based concentrating solar water heating system can be observed in Khullar et al.’s reports [83]. They thought that a great amount of emission reductions and energy savings could be achieved when implementing a nanofluid-based concentrating solar collector. Chaji et al. [84] investigated the effects of particles’ content and the liquid’s flow rate on the efficiency of a small-scale flat plate collector with TiO_2_ nanofluids. They found the index of collector efficiency using TiO_2_ nanofluids was increased by 2.6 to 7% compared to base fluid based on the European Standard EN12975-2. Said et al. [85] used TiO_2_–H_2_O nanofluid as the working fluid to enhance the performance of a flat plate solar collector. They observed the nanofluids prepared could keep stable for more than 1 month. The results showed that compared to water base fluid, the energy efficiency can be increased by 76.6 vol.% loading and 0.5 kg/min flow rate, and the highest exergy efficiency of 16.9% could be achieved at these operating conditions.

The theoretical research on the performance of a solar collector using nanofluids has also been developed in recent years. Alim et al. [86] studied theoretically the entropy generation, heat transfer characteristics, and the pressure drop of Al_2_O_3_, CuO, SiO_2_, and TiO_2_ nanofluids in a flat plate solar collector under laminar flow. They found that all kinds of nanofluids can improve the performance while the friction factor was almost similar to that of water-based fluid. Faizal et al. [87] also carried out a numerical study on the performance of those four kinds of nanofluids in the solar collector. They observed that the energy savings of all the four kinds of nanofluids can exceed 20%, which would result in emission reductions of greenhouse gases.

The performance enhancement by nanofluids in solar collectors is generally attributed to two primary factors: the enhanced heat transfer characters and optical properties. Therefore, the optical properties of nanofluids in solar absorption system were also investigated by researchers. Said et al. [88] performed both experiment and analytical studies on the solar absorption performance of TiO_2_ and Al_2_O_3_ nanofluids. They used two volume fractions of 0.1 to 0.3 vol.% for the photosensitive property investigation. Some classical theories including Rayleigh, Maxwell–Garnett, and Lambert–Beer’s approaches were adopted in their analytical analysis. They concluded that the optical properties of TiO_2_ nanofluids were higher than that of Al_2_O_3_ nanofluids within the range of visible light for all particle loading. He et al. [89] compared the light–heat conversion efficiency of the TiO_2_–water and CNT–water nanofluids in an evacuated tube solar collector in both sunny and cloudy conditions. They observed that the increment of temperature of CNT–water nanofluid is higher than that of TiO_2_–water nanofluids, which indicated that the light–heat conversion characteristic of the former is better than the latter.

Said et al. [90] thought that most research was focused on the fundamental thermophysical and optical properties of nanofluids; the studies on some important factor for scattering and absorption including particle size, shape, and content as well as base fluid type were rarely found. To examine those factors, they carried out related research and observed that the particle size has little effect when below 20 nm, and particle content was directly proportional to the extinction coefficient. For the nanofluids containing 20 nm TiO_2_ nanoparticles, the transmissivity was almost zero for wavelengths ranging from 200 to 300 nm, but 71% for 400 nm and 88% for 900 nm respectively. They also suggested that the volume fraction of TiO_2_ nanoparticles should be below 0.1%, at which a much better result can be obtained.

Kim et al. [91] carried out a detailed theoretical research by using MWCNT, Al_2_O_3_, CuO, SiO_2_, and TiO_2_ nanofluids with PG (propylene glycol)–water (20:80) base fluid in a high-temperature U-tube solar collector. They observed the collector efficiency of the solar collector efficiency has distinctly positive correlation with thermal conductivity of nanoparticles added since it is in the sequence from greatest to least: MWCNT, CuO, Al_2_O_3_, TiO_2_, and SiO_2_ nanofluids. They also analyzed the emission reduction of CO_2_ and SO_2_ as well as the electricity and energy consumption worldwide. Their results support that nanofluids has great potential for energy saving and emission reduction. Due to their theoretical results have not considered the dispersion situation of different nanofluids, the actual performance is needed to be experimentally verified.

Coincidentally, a similar experimental study of flat plate solar collector using different nanofluids was carried out by Verma et al. [92]. The experimental results indicated that for only 0.75% particle volume loading and at 0.025 kg/s flow rate, the exergy efficiency of nanofluids compared to water is increased by 29.32, 21.46, 16.67, 10.86, 6.97, and 5.74%, respectively, for graphene, CuO, Al_2_O_3_, TiO_2_, and SiO_2_ nanofluids. Also, the drop in entropy generation followed this sequence. Their results also supported that the solar collector efficiency has a positive correlation with thermal conductivity of nanoparticles added.

However, there are also some researches giving different results. Mahian et al. [93] analyzed the performance of a minichannel-based solar collector using four different water nanofluids with Cu, Al_2_O_3_, TiO_2_, and SiO_2_ nanoparticles respectively. Their results showed the Al_2_O_3_ nanofluids exhibited the highest heat transfer coefficient while the lowest value belonged to SiO_2_–water nanofluids, but the outlet temperature followed this sequence: Cu > TiO_2_ > Al_2_O_3_ > SiO_2_ nanofluids. They also observed that entropy generation of TiO_2_–water is lower than that of Al_2_O_3_–water nanofluids despite of the thermal conductivity of the former is lower than the latter.

## Refrigeration

Nano-refrigerant is a special kind of nanofluid which consists of nanoparticles and refrigerant as well as the probable lubricant. Nano-refrigerant is a new generation of refrigerant for using compression or absorption refrigeration, air conditioning systems, heat pumps, etc. In recent years, many studies regarding nano-refrigerants have shown that adding nanoparticles into refrigerants or lubricant can achieve a better system performance and energy efficiency.

**Fig. 7:**
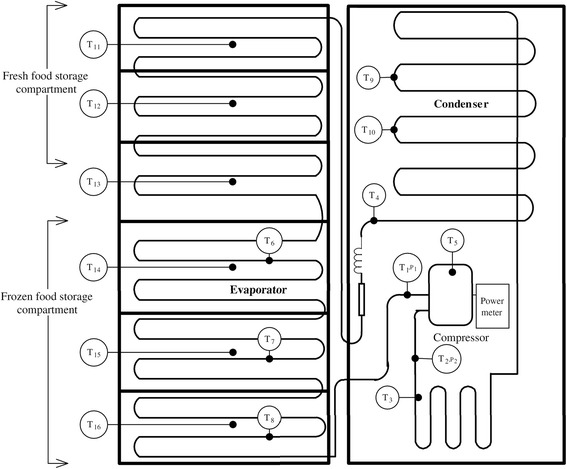
Schematic diagram of a domestic refrigerator with HFC134a, mineral oil and TiO_2_ nanoparticles [96]. Reproduced with permission from Elsevier

Table 2 shows summary of related studies on TiO_2_ nanoparticle-based nano-refrigerants. It can be seen that the TiO_2_ nanoparticles can work normally and safely with many kinds of refrigerants, including R134a, R600a, R436a, R436b, R141b, R123, R12, R22, and R410a. It can be seen that most results showed that adding TiO_2_ nanoparticles could bring benefits to the refrigeration system and the lubricating oil system, such as improving the performance [94], reducing the energy consumption [95–97], and the irreversibility [98]. Also, some research focused on the heat transfer [99, 100] and pressure drop [101] of the nano-refrigerant system to investigate the effect mechanism of the nanoparticles. Li et al. [102] investigated the coefficient of performance (COP) of the refrigeration system for both the cooling cycle and heating cycle, and the results showed that adding TiO_2_ nanoparticle would lead to a slight decrease in COP of the cooling cycle but a significant increase in COP of the heating cycle based on the power consumption of the compression. Bi et al. [96] experimentally investigated the reliability and performance of a domestic refrigerator with HFC134a as refrigerant and Mineral oil with TiO_2_ nanoparticles mixtures as lubricant.﻿ As illustrated in Fig. 7, the system main consists of fresh food storage room and frozen food storage room as well as refrigeration system pipelines. The results showed that the system TiO_2_ nanoparticles works normally and safely and adding 0.1 wt.% TiO_2_ nanoparticles can reduce 26.1% energy consumption while particle type has little effect on performance﻿.

**Table 2:** Summary of related studies on TiO_2_ nanoparticle-based nano-refrigerants

Researchers	Refrigerant	Nanoparticle	Lubricant	Particle size (nm)	Main finding
Bobbo et al. (2010) [94]	R134a	TiO_2_ (0.5 g/L)	POE (SW32)	21	(a) Adding TiO_2_ nanoparticles in SW32 oil showed the best performance as compared with the pure SW32 and single-wall carbon nano-horns/SW32 oil mixtures.
Mahbubul et al. (2011) [101]	R123	TiO_2_ (0.5 to 2 vol.%)	–	21	(a) The pressure drop increased with the increase of the particle volume fractions and vapor quality as well as the decrease of temperature.
Trisaksri and Wongwises (2009) [99]	R141b	TiO_2_ (0.01 to 0.05 vol.%)	–	21	(a) Nucleate pool boiling heat transfer performance was deteriorated with the increase of particle loading, especially at high heat fluxes.
Bi et al. (2007) [95]	R134a	TiO_2_ (10 mg/L)	Mineral oil	50	(a) Using nano-refrigerant could reduce the energy consumption of the system by 7.43%.
Bi et al. (2008) [96]	R134a	TiO_2_ (0.1 wt.%)	Mineral oil	50	(a) Adding 0.1 wt.% TiO_2_ nanoparticles can reduce 26.1% less energy consumption and particle type has little effect.
Bi et al. (2011) [97]	R600a	TiO_2_ (0.5 g/L)	–	50	(a) TiO_2_-R600a nano-refrigerant could work in the refrigerator normally and safely. (b) The refrigerator performance was better and 9.6% energy saved with 0.5 g/L TiO_2_-R600a nano-refrigerant.
Sabareesh et al. (2012) [100]	R12	TiO_2_ (0.01 vol.%)	Mineral oil	30/40	(a) An optimum volume fraction of 0.01% was found, at which the average heat transfer rate was increased by 3.6%, average compressor work was reduced by 11%, and COP was increased by 17%.
Padmanabhan and Palanisamy (2012) [98]	R134a, R436A, R436B	TiO_2_ (0.1 g/L)	Mineral oil	–	(a) TiO_2_ nanoparticles worked normally and safely with the three kinds of refrigerants/lubricant. (b) TiO_2_ nanoparticles can reduce the irreversibility of VCRS. Refrigerant R436A and R436B with MO + TiO_2_ as a lubricant obtained the best performance.
Javadi and Saidur (2015) [103]	R134a	TiO_2_ (0.1 wt.%)	Mineral oil	–	(a) Adding 0.1% of TiO_2_ nanoparticles to mineral oil-R134a resulted in the maximum energy savings of 25%. (b) An emission reduction of more than 7 million tons of Coz= by year of 2030 can be obtained in Malaysia.
Li et al. (2015) [102]	R22	TiO_2_ (5 wt.%)	–	–	(a) Adding TiO_2_ nanoparticle decreased COP of the cooling cycle slightly but increased COP of the heating cycle significantly due to the power consumptions of compression.
Chang and Wang (2016) [13]	R141b	TiO_2_ (0.0001% to 0.01 vol.%)	–	50–70	(a) The lowest concentration (0.0001%) TiO_2_ nano-refrigerant achieved the best performance (increased by 30%) with ultrasonic vibration.
Tazarv et al. (2016) [14]	R141b	TiO_2_ (0.01 and 0.03%)	–	30	(a) Convective heat transfer coefficient was greatly improved by adding TiO_2_ nanoparticles. (b) Low mass flux leads to a significant enhancement in heat transfer coefficient of TiO_2_ nano-refrigerant owing to the nanoparticle deposition.
Lin et al. (2017) [15]	R141b		NM56	60	(a) The suspending ratio of nanolubricant–refrigerant declined with the running time. (b) Lower particle loading, lower heating, or cooling temperature can reduce the degradation speed.

In addition, there is likewise a forward-looking study on the effect on the environment. Javadi and Saidur [103] observed that adding 0.1% of TiO_2_ nanoparticles to mineral oil-R134a could result in the maximum energy savings of 25% and reduce the CO_2_ emission by 7 million tons by year of 2030 in Malaysia.

It can be seen from Table 2 that the amounts of nanoparticles used in refrigerants were very low as below 0.1% [94–103], which can prevent clogging by the possible sedimentation of nanoparticles. However, although all results seemed positive, the long-term performance of the refrigeration system using nano-refrigerants is a great challenge.

**Fig. 8:**
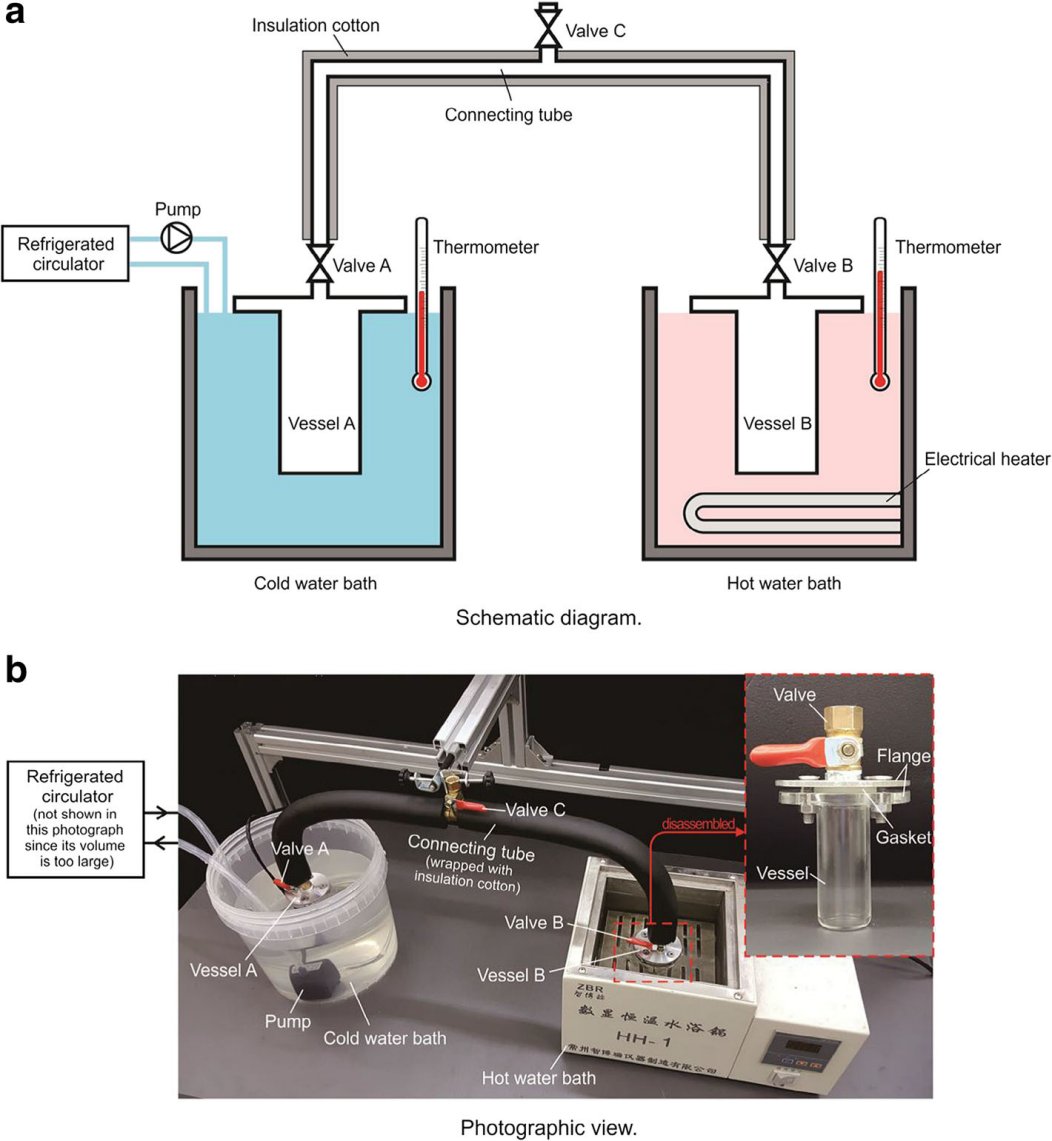
**a**, **b** Experimental setup for condensation–evaporation alternation [15]. Reproduced with permission from Elsevier

Lin et al. [15] investigated the suspending ratio of (0.1 to 1%) nanolubricant–refrigerant after continuous alternation processes of condensation and evaporation. The schematic diagram and photographic view of their experimental system is shown in Fig. 8. They found that the degradation ratio was 28 to 73% after 20 times’ alternate operations. Also, they found lower particle loading can reduce the degradation speed. It can be concluded that the longtime performance of nano-refrigerant system is the essential step for further application in nano-refrigeration system.

## Energy Storage

The storage of latent heat is through the most efficient mean of storing thermal energy. The conventional PCMs have a shortcoming of inadequate heat transfer performance which can reduce the rate of storing and releasing thermal energy. Therefore, some researchers have studied the method of improving the heat transfer performance by adding nanoparticles into PCMs.

Usages of PCMs mainly include energy storage of heating or cooling capacity. Research on cool storage application of TiO_2_ nano-PCMs is relatively rare. Liu et al. [104] find that thermal conductivity of saturated BaCl_2_ aqueous solution increases remarkably when adding a small amount of TiO_2_ nanoparticles. They found the thermal conductivity was increased by 15.65% as the TiO_2_ nanoparticle volume fraction was 1.13% at temperature of 15 °C. They thought this nanofluid is a good phase change materials (PCMs) with higher cool storage/supply capacity and rate compared with its base fluids, which exhibited good potential for being applied to cool storage as a substitute for conventional PCMs.

Another similar study was conducted by He et al. [105]. They also found the thermal conductivity of saturated BaCl_2_ aqueous solution can be distinctly enhanced by 12.76% when adding a small amount of TiO_2_ nanoparticles at −5 °C. Although decreases in the latent heat and specific heat and an increase in viscosity were found, those varieties have little effect on the cool storage system since the supercooling degree is reduced by 84.92%. They also thought that TiO_2_-saturated BaCl_2_ aqueous solution is suitable for low-temperature energy storage industries.

**Fig. 9:**
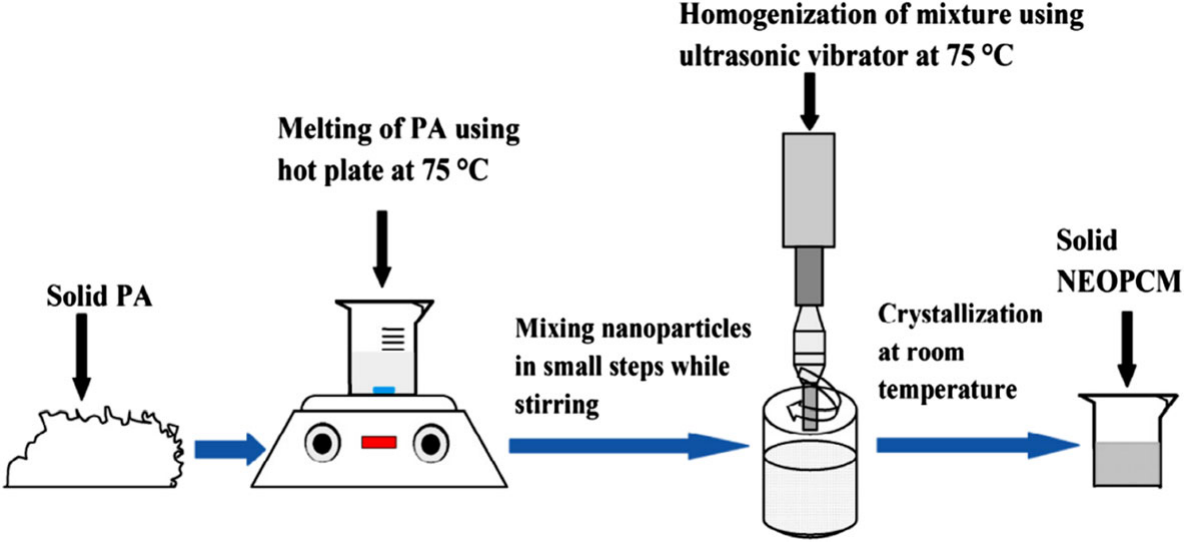
Preparation steps of PA–TiO_2_ composites [106]. Reproduced with permission from Elsevier

Studies on cool storage of TiO_2_ nano-PCMs are in the minority, while most PCM applications focus on the heat storage. Table 3 shows a brief summary on the thermal conductivities and the latent heat of TiO_2_ nano-PCMs for thermal storage applications in existing literatures. Sharma et al. [106] prepared a composite of palmitic acid (PA) and TiO_2_ nanoparticles with SDBS as dispersant for thermal energy storage application. The preparation steps of PA–TiO_2_ composites are shown in Fig. 9. It can be observed that the dispersion methods including adding surfactant, stirring, and ultrasonic vibration were implemented under the condition that the temperature of the base PA is above the melting temperature to confirm its liquid state.

**Table 3:** Summary on thermal conductivities and the latent heat of TiO_2_ nano-PCMs

Researchers	Composite	Thermal conductivity (W/m k)		Latent heat (kJ/kg)	
		Base PCM	Nano-PCM	Base PCM	Nano-PCM
Sharma et al. [106]	Palmitic acid/5% TiO_2_	0.194	0.35	–	180.03
Harikrishnan et al. [107]	Stearic acid/0.3% TiO_2_	0.19	0.31	131	127
Harikrishnan et al. [108]	(SA + LA)/1% TiO_2_	0.19	0.27	173.98	173.22
Motahar et al. [109]	n-octadecane/5% TiO_2_	0.45	0.57	–	–
Wang et al. [111]	Paraffin/0.7% TiO_2_	0.22	0.23	168	194
	Paraffin/7% TiO_2_	0.22	0.25	165	150

Their results showed that the thermal conductivity increased by 12.7, 20.6, 46.6, and 80% when the mass fractions of TiO_2_ nanoparticle were 0.5, 1, 3, and 5%, respectively. And they considered this PCM could be a good candidate as potential solar thermal energy storage materials due to its high latent heat and thermal reliability of palmitic acid. Harikrishnan et al. [107] dispersed TiO_2_ nanoparticles into PCM stearic acid and found this composite can accelerate the melting and solidification rates due to the enhanced heat transfer performance. They also observed that the addition of 0.3% nano-TiO_2_ nanoparticles can increase the thermal conductivity of stearic acid by approximately 63%. In their another research [108], they used stearic acid and lauric as base PCM and found an increment of 42% in thermal conductivity and a reduction of only 2% of latent heat of fusion. Motahar et al. [109] dispersed the TiO_2_ nanoparticles into organic PCM n-octadecane and found that the maximum enhancements of thermal conductivity in solid and liquid phases occurred at 3 and 4 wt.%, respectively. Moreover, the maximum average thermal conductivity enhancement for both phases was 26.6% when loading 5 wt.% nanoparticles.

Another experimental research focusing on the solidification process of PCM containing TiO_2_ nanoparticles was also performed by Motahar et al. [110]. They observed that the rheological behavior of liquid PCM–TiO_2_ at higher loading tends to Bingham fluids so that their solidification experiments were performed within 0–2.17 Bingham numbers. The results showed that the addition of TiO_2_ nanoparticles can enhance the thermal conduction process and hence increase the solidified volume. For particle mass loading of 1, 2, and 4%, the solidified volume fraction was increased by 7, 9, and 18%, respectively. At last, they proposed a universal correlation to predict the solidified volume fraction as a function of Fourier number, Rayleigh number, solid Stefan number, Bingham number, and particle loading.

Most of the results showed that when adding TiO_2_ nanoparticles, the thermal conductivity of PCMs can be greatly increased, while the latent heat will be decreased slightly, which is probably as a result of the thermal conductivity of nanoparticles which is much larger than the base composite, while the nanoparticles will not take part in the phase changing process as the base composite. However, in some case, both of the thermal conductivity and latent heat capacity of PCMs were considered to be elevated. Wang et al. [111] prepared nano-PCMs by adding TiO_2_ nanoparticles into paraffin. They found the addition of TiO_2_ nanoparticles can change the phase transition temperature and latent heat capacity of paraffin. The phase transition temperature dropped with <1% loading, while increased with >2% loading of particles. The latent heat increased firstly and then decreased as the loading of particles increase. And the turning concentration is 0.7 wt.%, at which a maximum latent heat capacity can be achieved. While the thermal conductivity of the nano-PCMs increased monotonously with the loading of TiO_2_ nanoparticles. When the loading of TiO_2_ nanoparticles reached 7 wt.%, the thermal conductivity was increased by 13% but the latent heat was reduced by 9%.

## Heat Pipes

The characteristics of boiling heat transfer and critical heat flux enhancement of nanofluids can be utilized in the heat pipe to improve its performance and broaden the application range. And some numerical results [112, 113] have shown that for thermosyphon heat pipe, using the nanofluid could achieve a better heat transfer characteristics. Also, some researchers have carried out related research using TiO_2_ nanofluids.

Zhou et al. [114] tested gravity heat pipes filled with DI water and TiO_2_ nanofluids, where the concentration and filling ratio of nanoparticles were varied and the initial temperature distribution was given. The result indicated that the heat pipes filled with nanofluids had a lower start-up temperature and a shorter start-up time in evaporation section under the condition of a water bath. And the biggest temperature drop between the evaporation section and the condensation section for heat pipes filled with TiO_2_ nanofluids was lower than those filled with DI water. The start-up time of heat pipes with filling ratios ranged between 50 and 70% in the evaporation section increased with the increase of the filling ratio and heating temperature, but the small inclination angle had a negative effect on the start-up performance.

Saleh et al. [65] collected data from different nanofluid experiments, where particle volume loading was up to 1.0% and the temperature of measurements ranged from 10 to 60 °C. They discovered that these data agreed with the classical Brownian motion theoretical model. They also investigated experimentally the effect of nanofluids on the thermal performance of heat pipes by measuring the wall temperature and thermal resistance distributions between the evaporation and condensation section. They found that distilled water and nanofluids achieved the best heat transfer performance when the inclination was set to 45° and the charge volume ratio of working fluid was 60%.

**Fig. 10:**
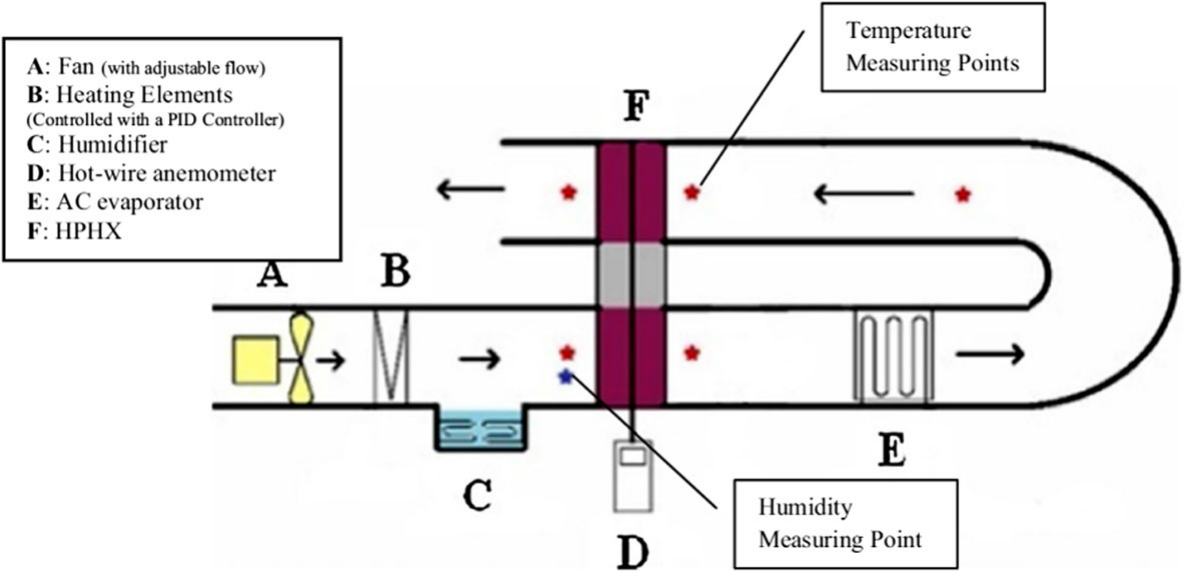
Schematic of the experimental apparatus [115]. Reproduced with permission from Springer

In 2015, Monirimanesh et al. [115] designed a thermosyphon-type heat pipe heat exchanger (HPHX) using TiO_2_ nanofluids as the working fluid to save energy in an air conditioning system. Their experimental apparatus was constructed as shown in Fig. 10. They establish a pre-cooling and pre-heating device to produce altered conditions of the inlet air for investigating the performance of HPHX. The evaporator and condenser section of the HPHX functioned as a pre-cooler and reheating coil for the air conditioning system respectively. They also employed an electric heater and electric boiler to supply heat and steam into the entered fresh air by a fan for the purpose of simulating the hot and humid climate. Their results showed that using TiO_2_ nanofluids and increasing the HPHX number of rows could make a part of air condensed on the evaporator fin, which could enhance the energy in the pre-cooling section. The use of 3 wt.% TiO_2_–methanol nanofluids in a four-row HPHX could achieve the highest energy savings ranging from 30.6 to 32.8% when the inlet air under the properties of 45 °C and 50–74% relative humidity. Based on a comprehensive consideration of the main purpose of supplying the energy required for reheating, they suggested that 2 wt.% TiO_2_–methanol nanofluid for the four-row HPHX would have been adequate and more economical.

## Mass Transfer

The mass transfer of nanofluids is another important application aspect of TiO_2_ nanofluids. Current research has shown that TiO_2_ nanofluids can be used to enhance the absorption process of CO_2_ and NH3 as well as the mass transfer coefficient of electrolyte fluids.

Li et al. [116] prepared stable *N*-methyldiethanolamine (MDEA)-based nanofluids to strengthen the absorption performance of CO_2_ in the MDEA solution. The CO_2_ absorption characteristics in the gas/liquid interface of nanofluids were investigated by measuring the absolute pressure drop of gas. The mass fraction of MDEA was 50%. And they used two particle mass fractions of 0.1 and 0.4%. The results showed that the CO_2_ absorption rate increases with increasing temperature and it is enhanced by the added nanoparticles. However, at 20 and 30 °C, the enhancement caused by the mass fraction of nanoparticle (0.1 and 0.4%) reduced gradually. The effective absorption ratio varied from 1.03 to 1.14. Also, CO_2_ bubble absorption ratio increased with the increase of nanoparticle mass fraction.

**Fig. 11:**
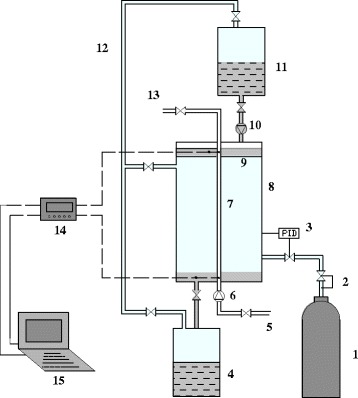
Schematic diagram of the experimental system for NH_3_–H_2_O nanofluid falling film absorption [117]. *1* NH3 vessel, *2* decompression valve, *3* constant pressure controller, *4*, *11* container of solution, *5* inlet of cooling water, *6*, *10* constant flow controller, *7* falling film tube, *8* visible absorber body, *9* solution distributor, *12* tubes for balancing pressure, *13* outlet of cooling water, *14* HP data acquisition instrument, *15* computer

Yang [117] prepared stable TiO_2_ nanofluids without adding dispersant and then carried out a comparative experiment on the falling film performance of absorption of ammonia gas by nanofluids and pure water. The schematic diagram of the experimental system for NH_3_–H_2_O nanofluid falling film absorption is shown in Fig. 11. They found that the absorption rate of ammonia gas can be increased by 10% when adding anatase TiO_2_ nanofluids. Wu [118] used the similar experimental device but changed the falling film tube of Fig. 11 into a fin tube. He investigated the effect of rutile TiO_2_ nanofluids on the ammonia absorption performance of falling film outside a fin tube. The result showed that the combined use of zigzag tubule and TiO_2_ nanofluids can strengthen the ammonia–water falling film absorption and the maximum increment can reach 60.8%.

Beiki et al. [119] investigated experimentally the turbulent mass transfer characteristics of TiO_2_ and γ-Al_2_O_3_ electrolyte nanofluids in a circular tube. The results showed that adding 0.015 vol.% TiO_2_ and 0.01 vol.% γ-Al_2_O_3_ could bring an increase in mass transfer coefficient of the electrolyte solution by 18 and 10%, respectively. They found that the enhancement ratio was independent of Reynolds number. The mass transfer coefficients increased firstly and then decreased as the nanoparticle loading increase. They attributed the cause of the existing of optimal particles’ loading to the clustering of nanoparticles and forming bigger agglomerates with smaller Brownian velocity when exceeding the optimum loading.

## Coolant of Milling

As a coolant, nanofluids’ heat transfer enhancement characteristic can improve the cooling performance [120]. Moreover, when nanofluids are used for milling, another characteristic of nanofluids viz. enhancement in wear resistance can also play an important role in extending the lifetime of the milling tool.

Yogeswaran et al. [121] investigated experimentally the effects of coolant of TiO_2_–EG nanofluid on the tool wear and workpiece temperature at the various milling conditions when used for milling a stainless steel AISI 304. The milling tool was made of a TiN-coated carbide insert. The results showed that comparing to pure base fluid, the workpiece temperature was reduced by 30% when using the nanofluid as coolant. The tool wear from milling using the EG-based TiO_2_ nano-coolant is much less than using the normal commercial coolant because the nanofluids can reduce the heat penetrating into the inserts. And the tool life is increased as a result of the nanoparticles reduces the damage on the edge of the tool.

**Fig. 12:**
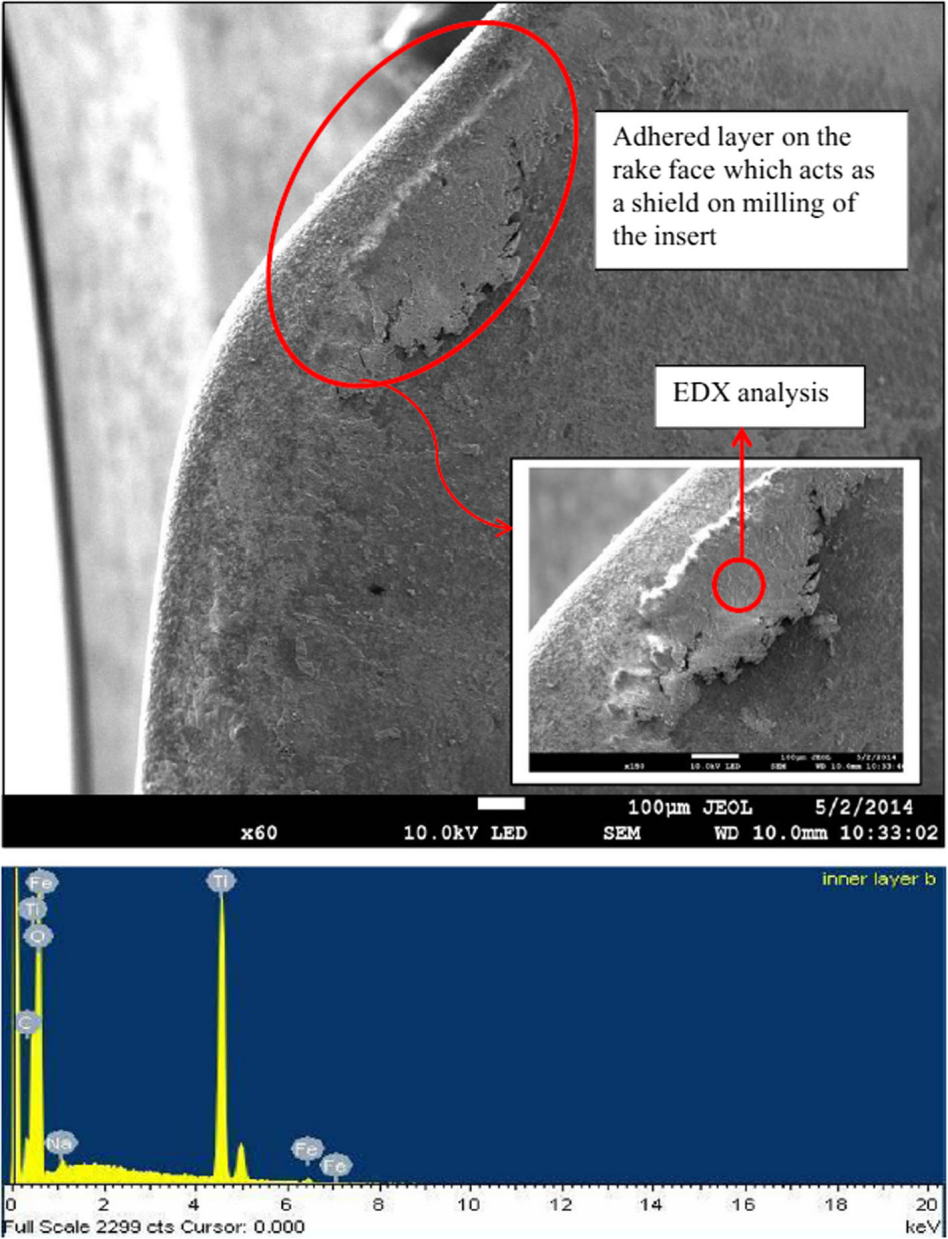
SEM and EDX of cutting edge [122]. At a cutting speed of 1500 rpm, feed rate of 0.02-mm tooth, and axial depth of 0.1 mm using nanoparticle-based coolant at a cutting distance of 180 mm (×60 magnification). Reproduced with permission from Springer

Muthusamy et al. [122] also compared the efficiency of nanoparticle-based coolant (TiO_2_–EG) and conventional water-soluble coolant on the tool life and wear performance of a TiN-coated carbide insert in the end-milling process of AISI304 stainless steel. The results showed that using TiO_2_–EG nanofluid as coolant could increase the tool life from 32.67 to 54.9 min (increased by 40.55%) comparing to that using TiO_2_–EG nanofluid as coolant instead of water-soluble coolant. They attributed the cause to a Ti nanoparticle layer on the edge of the insert formed during the milling process when using TiO_2_–EG nanofluid, which can be proved from the SEM and EDX of cutting edge as shown in Fig. 12. When using nanofluids as coolant, the oxidation still occurred despite the cutting temperature was reduced at the interface of the tool and workpiece since it can be found from Fig. 12 the O peak on the EDX spectrum. The hard oxidation layer was formed due to the entering of oxygen from TiO_2_–EG nanofluid into the tool–workpiece interface. Then, the hard oxidation layer can protect the tool from micro-cracking and chipping wear because it could not be easily detached despite under the severe impact of the milling force and took parts of the tool surface from the workpiece.

## Challenges and Future Works

### Challenges

The above energy-related examples have exhibited the extensive application prospect and excellent properties of TiO_2_ nanofluids. Although in some cases, especially in heat transfer applications, the heat transfer performance of TiO_2_ nanofluids are not better than that of Ag, Cu, and CNT nanofluids, TiO_2_ nanofluid is also a good choice due to their comprehensive properties for instance better dispersion and chemical stability, security, and economy.

Although TiO_2_ nanofluids have showed great enhancement in heat transfer of solar collectors, refrigeration, energy storage, heat pipes, and coolant of milling, the investigations on the performances including dispersion stability and heat transfer performance after running operations are in great lack. Most dispersion stability studies are in static conditions, but it is important that the nanofluids prepared should be treated in the practical application conditions to examine the dynamic cycle stability and the sustainability of both system performance and components of nanofluids.

Generally, the biggest downside in application of nanofluids is the sedimentation and degeneration of nanoparticles after long running which makes the long-term performances of nanofluid system challenged. Some researchers have proposed a new idea and a novel method to re-disperse the aggregates in real time of the running system [123]. However, the concrete effect of the device has to be verified experimentally, and then, the design and location of the re-dispersion device needs to be improved. The surfactant is expected to have positive effect on the re-dispersion characteristic of aggregates. However, one of the biggest flaws in using surfactants is the occurrence of foaming when the fluids are under flowing or heating conditions which would have adverse impacts on the heat or mass transfer application of nanofluids. This defect suggests the amount of surfactant employed in the nanofluids should be limited.

Another great limitation in application of nanofluid is the increase in pumping power and pressure drop of nanofluids, which is essential for the high-quality application of solar collectors, refrigeration, and heat pipes. For instance, Sajadi and Kazemi [124] found the proportional increase in pressure drop of TiO_2_ nanofluids is higher than that of heat transfer coefficient. While Teng et al. [125] found the pressure drop proportion of TiO_2_ nanofluids for turbulent flow is lower than that for laminar flow. Therefore, if the extra energy consumption by the increased viscosity of nanofluids exceeds the benefit from the heat or mass transfer enhancement, there will be no application prospect. The most extreme case is when a large amount of agglomerations emerge, the pumping power and pressure drop of nanofluids will be greatly increased, which might lead to serious impact on system performance. Moreover, based on the similarity principle in heat transfer study, for instance in forced convection process, Nusselt number is determined by Reynolds number and Prandtl number, different thermal conductivity and viscosity will induce different Nu even though for the same experimental heat transfer coefficient. Therefore, the properties of nanofluids are essential for quantitative study in those application fields.

### Future Works

As a widely used material in considerable fields, TiO_2_ has been explored several hundred years, and its nanofluidic form is also firmly worth studying and expected to make greater contributions owing to the outstanding physical and chemical properties. This paper provides a summary of the research outcomes of TiO_2_ nanofluids up to now, including the preparation and stability of TiO_2_ as well as three vital properties of TiO_2_ nanofluids. It can be concluded that TiO_2_ nanofluids show very comprehensive applications in heat transfer or other energy fields due to their good dispersion stability in both hydrophilic and lipophilic liquids, nontoxic and non-corrosive natures, chemical stability, lower price, and good appearance. Therefore, TiO_2_ nanofluid is thought as one of the closest kinds to practical industrial application environment because of their better dispersion and chemical stability, security, and economy.

However, although TiO_2_ nanofluids have shown enormously exciting potential applications, before commercialization of nanofluids, some urgent problems are summarized as follows:

Firstly, acquiring high-quantity nanofluid with outstanding long-term and high-temperature stability is the fundamental of the entire research since in any practical application, it is essential to have a stable suspension.

Secondly, the way to enhance and keep the stability of nanofluids in real time is a key issue in the actual use since the sedimentations of nanoparticles seem inevitable after a long-term running. The method to re-disperse the aggregation of nanoparticles in real time by adding some dispersion device in the system with functions of ultrasound or agitation might be a useful option [123].

Thirdly, although the surfactants were used to improve the dispersion and adhesion performance of nanoparticles in liquid, the effect of surfactants on the physical properties and system performance needs to be investigated. The amount of surfactants should be investigated experimentally owing to the positive and negative effects of surfactants.

Fourth, the pumping power or pressure drop of nanofluids is another challenge for the engineering application. Using nanofluids with higher viscosity than base fluids will induce a higher pressure drop and hence needs more pumping power [125]. The method to achieve higher heat transfer coefficient and lower pressure drop needs to be further studied.

Fifth, the waste management of the invalid nanofluids should also be considered when applying them to industrial systems. The impact on the environment by the nanofluids restricts many kinds of nanofluids containing heavy metal, toxic substance, or other hazardous substances. The super whiteness dyeing behavior of TiO_2_ nanofluids should also be noticed to prevent the environment getting contaminated.

Sixth, although some studies have analyzed the entropy generation in tubes [126], microchannels [127], sheet, and other types of flow [128, 129], the entropy generation characteristic of nanofluid in the full system is actually the most important parameter for the full-system application or designing.

Last but not least, there is lack of evaluation index on the performance of nanofluids, especially on the stability, adhesion, and property sustainability of nanofluids. There is no unified indicator to evaluate the stability and adhesion of nanofluids. The uniform evaluation indexes on the different properties of nanofluids are needed [130].

The above problems are urgently needed to solve for the further application of TiO_2_ nanofluids, which point out the directions of the future works in this field. It is believed that these problems and challenges will be solved or reduced with the development of nanofluid technology in the future.

## Conclusions

This second part of the review summarizes recent research on application of TiO_2_ nanofluids and identifies the challenges and opportunities for the further exploration of TiO_2_ nanofluids. It can be concluded that although particle loading has exhibited a positive correlation with thermal conductivity of nanofluids, the effects of other factors including particle shape, size, base fluid type, temperature, surfactant, and sonication are unified. Even for particle loading effect, the intensities of growth in thermal conductivity differ widely for different samples. TiO_2_ nanofluids have shown good applications in many energy-related filed. However, the indeterminacy of long-term performances for both nanofluid and system and the increment in pressure drop are needed to investigate for further application. The forecast research hotspots are regarded as the long-term and high-temperature stability and re-disperse the aggregation of nanoparticles in real-time system, the required amount of surfactants, the heat transfer and pumping power characteristics, and the evaluation index on the stability, adhesion, and property sustainability of nanofluids.
